# Crab bioturbation alters the community assemblies of abundant and rare bacteria on an intertidal wetland in the Yellow River estuary

**DOI:** 10.3389/fmicb.2025.1521363

**Published:** 2025-01-31

**Authors:** Zhikang Wang, Yongqi Wang, Jisong Yang, Junfen Yan, Kaixin Yang, Zhonghua Ren, Wei Wang, Yang He, Min Li, Junfei Zhan, Bo Guan, Xuehong Wang, Yunzhao Li, Di Zhou, Buli Cui, Junbao Yu

**Affiliations:** ^1^Institute for Advanced Study in Coastal Ecology, School of Resources and Environmental Engineering, Ludong University, Yantai, China; ^2^Dongying Academy of Agricultural Sciences, Dongying, China

**Keywords:** crab bioturbation, community assembly, co-occurrence network, rare bacteria, coastal wetland

## Abstract

**Introduction:**

Revealing assembly patterns of abundant and rare bacteria is pivotal for comprehending the responses of soil bacterial community to environmental changes. Crabs exert significant impacts on soil environments through their frequent burrowing activities in intertidal wetlands. However, there remains a paucity of knowledge regarding the influencing mechanism of crab bioturbation on community assemblies of abundant and rare bacteria.

**Methods:**

We delved into community structures, co-occurrence networks, and assembly processes of abundant and rare bacteria within crab-bioturbated soils (encompassing burrows and mounds) across an intertidal wetland.

**Results and discussion:**

The compositions and diversities of abundant and rare subcommunities were notably altered in crab-bioturbated soils. Moreover, the co-occurrence network analysis unveiled that crab bioturbation substantially modified the interaction patterns of rare bacteria, whereas its influence on abundant bacteria was comparatively minor. Furthermore, we discovered that the assembly processes of abundant subcommunities were primarily influenced by stochastic processes, while rare subcommunity assemblies were collectively shaped by both stochastic and deterministic processes. In conclusion, our study elucidates the mechanism by which crab bioturbation mediates the distinct assembly processes of abundant and rare subcommunities, and underscores the importance of considering rare bacteria when evaluating the ecological functions of intertidal wetlands.

## 1 Introduction

Coastal wetlands, situated between terrestrial and marine ecosystems, have garnered increasing attention due to their crucial ecosystem functions, including carbon sequestration, global climate change regulation, and biodiversity preservation (Murray et al., 2019; Wang F.M. et al., 2020; Alongi, 2023). Soil microorganisms serve as vital engines driving fundamental functions such as element biogeochemical cycles, contaminant degradations, and greenhouse gas emissions (An et al., 2019; Zilius et al., 2020). In various natural ecosystems, it now widely recognized that the assembly of soil microorganisms into communities is governed by stochastic processes (e.g., homogenizing dispersal, dispersal limitation, and undominated process) and deterministic processes (e.g., homogeneous/heterogeneous selection) (Vellend, 2010; Hanson et al., 2012; Nemergut et al., 2013; Zhou and Ning, 2017). Numerous studies in coastal wetlands have demonstrated that soil environmental factors affect microbial community assembly by regulating the balance between stochastic and deterministic processes. For instance, the assembly of soil bacterial communities is controlled by stochastic processes in the early formation stage of salt marshes, and then becomes dominated by deterministic processes as soil salinity and organic matter increase in mature salt marshes (Dini-Andreote et al., 2015). Moreover, previous researches have revealed that the assembly of bacterial communities exhibits a progressive transition from stochastic to deterministic processes along a gradient from low tidal flats to the supratidal zone, dirven by changes in soil factors such as soil moisture, salinity, and nutrients (Yao et al., 2019; Huang et al., 2022).

Generally, the microorganisms exhibit unbalanced distributions within a local community, characterized by the coexistence of a few high-abundance taxa and the majority of low-abundance but highly diverse rare taxa (Pedrós-Alió, 2012; Jiao et al., 2017; Jia et al., 2018). The abundant subcommunity is widespread and often responsible for maintaining the stability of the entire bacterial community, even in the face of dramatic environmental fluctuations (Delgado-Baquerizo et al., 2017; Jiao et al., 2019; Yang et al., 2022). Conversely, the rare subcommunity is susceptible to environmental filtering and exists within a narrow niche breadth, yet it provides limitless reservoirs of genetic and functional diversity (Jousset et al., 2017; Jiang et al., 2020; Wang et al., 2023). Given these findings, it seems that the abundant and rare microbial subcommunities may undergo different assembly processes due to their differing environmental tolerance and niche breadth. Furthermore, some recent studies have pointed out that rare bacteria can greatly affect the stochastic-deterministicity balance in wetland ecosystems (Gao et al., 2020; Ma et al., 2023). Therefore, unveiling the relative contributions and assemblies of abundant and rare bacteria is critical for understanding the mechanisms involved in bacterial biodiversity formation and maintenance in coastal wetlands.

Crabs are considered as important ecological engineers with frequent activities in intertidal wetlands, which greatly change soil microtopography, organic carbon redistribution and biogeochemical processes (Tomotsune et al., 2020; Xiao et al., 2020; Forgeron et al., 2021; Xiao et al., 2024). Crab burrows not only retain plant residues to accumulate soil nutrients but also promote greenhouse gas emissions through the decomposition of soil organic matter (Qiu et al., 2019; Xie et al., 2020; Chen et al., 2020; Agusto et al., 2022). It has been estimated that crab bioturbation could result in a loss of carbon sequestration totaling 0.35 Tg C·a^−1^ per year in global coastal wetlands (Guimond et al., 2020). Moreover, several reports have found that crab bioturbation reshapes soil bacterial community compositions and diversities in salt marshes (Cuellar-Gempeler and Leibold, 2018; Wu et al., 2020). In particular, some bacteria closely associated with soil nutrient biogeochemical cycles (e.g., cellulose-degrading, nitrogen-fixing, and sulfate-reducing bacteria) have higher abundances in burrow soils (Zilius et al., 2020; Tongununui et al., 2021; An et al., 2022), suggesting crab burrows were the hotspots of nutrient transformations. However, these studies were mainly focused on the effects of crab bioturbation on entire community structures and predominant microorganisms, there have been few investigations in rare microorganisms which may occupy important ecological niche in coastal wetland (Du et al., 2020; Gao et al., 2020). Moreover, few study focused on the effects of crab bioturbation on microbial community assembly, which may limit our understanding of the microbial ecosystem processes driven by crab bioturbation in coastal wetlands.

To answer these questions, we conducted a field investigation in the Yellow River estuary wetland to explore the effects of crab bioturbation on abundant and rare bacterial subcommunities and their assembly mechanisms, based on 16S rRNA high-throughput sequencing technology. We further endeavored to evaluate the relative importance of abundant and rare bacteria in community structures and co-occurrence networks during crab bioturbation in different salt marshes. The Yellow River estuary wetland is an important coastal wetland with high biodiversity and high-frequency crab activity (Wang X.H. et al., 2020; Xie et al., 2022). We addressed the following hypotheses: (1) Crab bioturbation alters the community structures and co-occurrence network of abundant and rare bacteria. (2) Abundant and rare subcommunity assemblies are governed by distinct assembly processes. (3) The relative contributions of assembly processes are influenced by crab bioturbation.

## 2 Materials and methods

### 2.1 Soil sampling and soil physicochemical properties analysis

**Figure 1:**
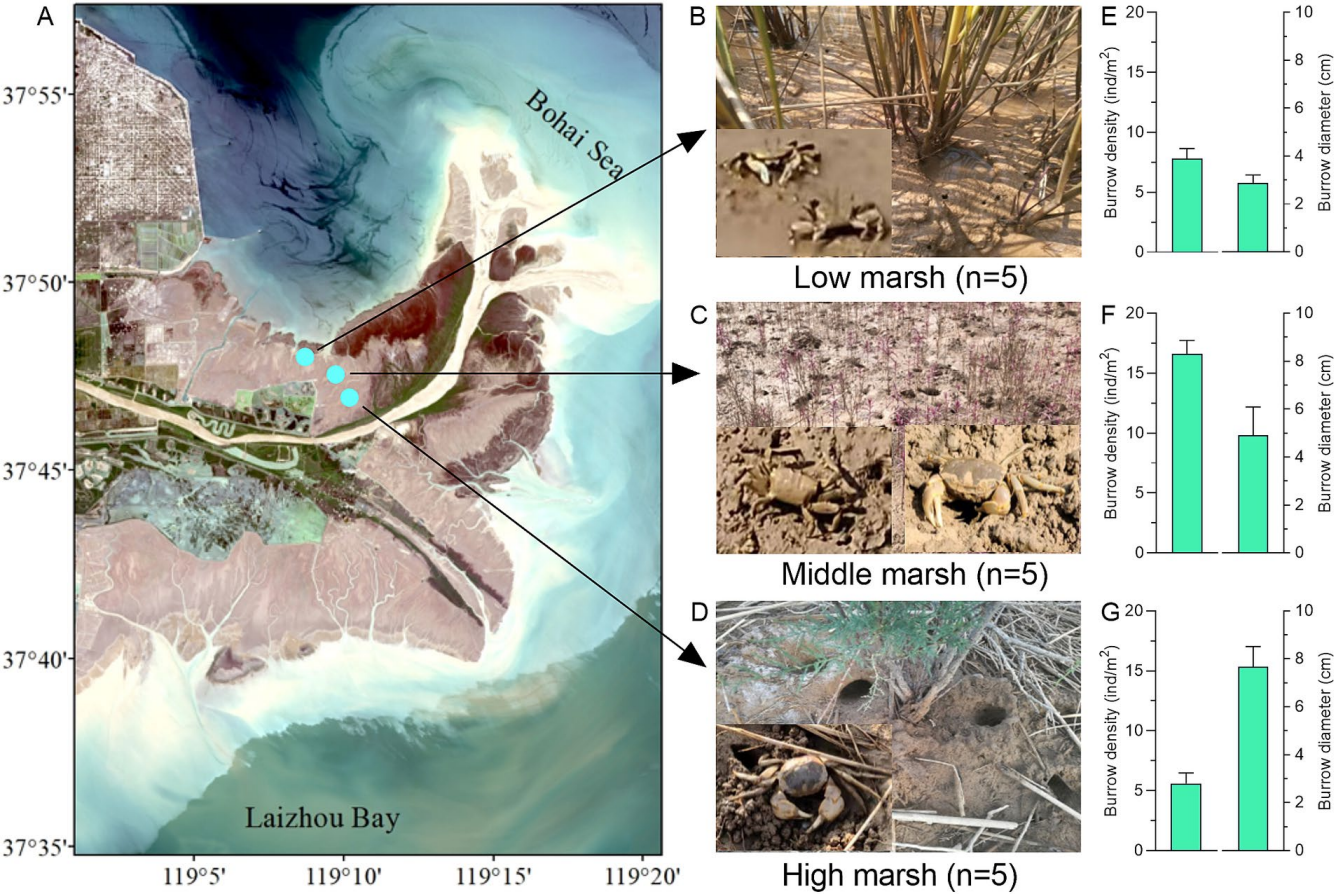
**(A)** Location of three salt marshes on a natural intertidal wetland in the Yellow River estuary wetland. Field investigation of crab burrowing bioturbation in low marsh **(B)**, middle marsh **(C)**, and high marsh **(D)**. The densities and diameters of crab burrows in low marsh **(E)**, middle marsh **(F)**, and high marsh **(G)**.

A Field investigation was conducted to study crab burrowing bioturbation in a natural intertidal salt marsh (37°46′—37°48′N, 119°9′—119°11′E) located in the Yellow River estuarine wetland, Eastern China (Figure 1A). The selected area, in which crab burrows and mounds are widely distributed, was partitioned into low marsh (LM), middle marsh (MM) and high marsh (HM). Through field observations, it was found that the dominant plants were *Spartina alterniflora* in LM, *Suaeda salsa* in MM, and *Tamarix chinensis* in HM, respectively (Figures 1B–D). Five sampling sites (1 m × 1 m) were randomly established in each marsh to count the density and diameter of crab burrows (diameter ≥ 2 cm).

The crab bioturbated soils (burrows and mounds) and controls were as far as possible from plant roots to attenuate the selection effects of different plants. Soil samples were collected from crab burrows (5–10 cm depths) and mounds (0–5 cm depths) as bioturbated samples, and non-bioturbated soils were used as controls at depths of 0–10 cm. Some soil samples were immediately sealed in sterile Eppendorf tubes and refrigerated for 16S rRNA high-throughput analysis, and the others were picked out residues and naturally air dried to grind through a 100-mesh sieve for measure soil physicochemical properties. We used standard soil test methods to measure six soil physicochemical properties (Lu, 2000), including soil moisture content (SMC), pH value, electrical conductivity (EC), soil organic carbon (SOC), total nitrogen (TN), and total phosphorus (TP). Briefly, SMC was determined by the weight loss after drying 20 g of wet soil at 105°C for 24 h. Soil pH and EC were determined using a pH meter and an electronic probe (with a soil-to-water ratio of 1:5). SOC, TN, and TP were determined using potassium dichromate heating oxidation-volumetric method, Kjeldahl nitrogen method, and Mo-Sb anti-spectrophotometric method, respectively.

### 2.2 16S rRNA PCR and Illumina MiSeq high-throughput sequencing

We extracted microbial DNA from 45 soil samples utilizing the E.Z.N.A.® soil DNA Kit (Omega Bio-Tek, Norcross, GA, USA). The final DNA concentration and purity were subsequently determined using a NanoDrop 2000 UV–vis spectrophotometer, and DNA quality was verified by 1% agarose gel electrophoresis. To identify bacteria species, we selected V4-V5 hypervariable regions to amplify the 16S rRNA from qualified DNA templates using barcoded primers 515F/907R (5′-GTGCCAGCMGCCGCGG-3′/5′-CCGTCAATTCMTTTRAGTTT-3′). The PCR products were purified with an AxyPrep DNA Gel Extraction Kit and then quantified using QuantiFluor™-ST (Promega, USA). The purified amplicons were pooled in equimolar concentrations and subjected to paired-end sequencing (2 × 300) on an Illumina MiSeq platform (Illumina, San Diego, CA, USA), adhering to the standard protocols by Majorbio Bio-Pharm Technology Co. Ltd. (Shanghai, China).

Raw fastq files underwent quality-filtering using Trimmomatic and were merged with FLASH software. Operational taxonomic units (OTUs) were identified using UPARSE (version 7.0.1090) with a 97% identity cutoff based on a novel ‘greedy’ algorithm. The selected sequences were then assigned to the SILVA project release version 138^1^ using the RDP classifier algorithm^2^ with a confidence threshold of 70%. Finally, the resulting OTUs were subjected to minimum normalized subsampling to generate a resampled OTU table for subsequent statistical analysis.

### 2.3 Definition of abundant and rare taxa

To evaluate the impact of crab bioturbation on soil bacterial community, we applied different cutoffs of relative abundance to distinguish between abundant and rare subcommunities. Consistent with previous studies (Dai et al., 2016; Chen et al., 2019), all OTUs were classified into six categories, including of always abundant taxa (AAT), conditionally abundant taxa (CAT), always rare taxa (ART), conditionally rare taxa (CRT), moderate taxa (MT), conditionally rare and abundant taxa (CRAT). For further comparative analyses, the AAT, CAT and CRAT were combined as abundant taxa (AT), ART and CRT were combined as rare taxa (RT).

### 2.4 Bacterial community assembly process analysis

The community assembly process was elucidated using the “ape,” “NST,” and “picante” packages in R (Ning et al., 2020). β-NTI (beta nearest taxon index) and Raup Crick index (RC_Bray_) were computed to represent phylogenetic and taxonomic diversity (Stegen et al., 2013; Stegen et al., 2015). Specifically, |β-NTI| > 2 indicates dominance of the deterministic process, while |β-NTI| < 2 suggested dominance of the stochastic process. β-NTI < −2 represents homogeneous selection (HoS), and β-NTI > 2 indicates heterogeneous selection (HeS). For |β-NTI| < 2, RC_Bray_ < −0.95 signifies homogenizing dispersal (HoD), while RC_Bray_ > 0.95 indicates dispersal limitation (DiL). When |β-NTI| < 2 and |RC_Bray_| < 0.95, it denotes undominated assembly (UnD), primarily consisting of weak selection, weak dispersal, diversification, and/or drift.

### 2.5 Bacterial co-occurrence network analysis

The empirical and random co-occurrence networks were constructed using “igraph” package in R. The selected OTUs of empirical networks should meet these standards: (i) the relative abundances ≥0.01%, (ii) the frequency of occurrences >60%, (iii) the absolute values of Pearson correlation coefficients >0.6 and *p* < 0.05. Random networks were generated by corresponding empirical networks with an identical number of nodes and links, and constructed with 1,000 simulations using the Erdös–Rényi model. Moreover, we used Gephi platform to visualize these co-occurrence networks based Fruchterman–Reingold layout algorithm. To characterize the small-world properties of co-occurrence networks, a set of topological properties, including average path length (APL), average clustering coefficient (ACC), modularity degree (MD), and small-world coefficient, were calculated. Furthermore, node-level topological properties (e.g., degree centrality, betweenness centrality, closeness centrality, module) were used to identify core modules and OTUs.

### 2.6 Data analyses

To ascertain statistically significant differences, one-way ANOVA in SPSS statistics (version 25) was used to calculate the soil physicochemical properties, OTU relative abundances (AT and RT), α-diversity indices (ACE and Shannon–Wiener), and bacterial community compositions. Furthermore, β-diversity was assessed using the Bray–Curtis dissimilarity metric via metaMDS in the “vegan” package, and the results were visualized through non-metric multidimensional scaling (NMDS) using the “ggplot2” package in R program (V.4.2.3). Additionally, correlations between β-nearest neighbor species classification indexes (β-NTIs) and environmental factors were analyzed using the Mantel test, and relationships between bacterial community structures and environmental factors were examined through Redundancy Analysis (RDA), both utilizing the “vegan” and “ggplot2” packages.

## 3 Results

### 3.1 The impact of crab bioturbation on soil physicochemical properties

The field investigation showed that the crab bioturbations in salt marshes were dominated by two crab species, *Macrophthalmus japonicus* and *Helice tientsinensis* (Figures 1B–D). The burrow densities and diameters were found to be 7.80 ± 0.84 ind/m^2^ and 2.88 ± 0.33 cm in low marsh (LM), 16.60 ± 1.14 ind/m^2^ and 4.91 ± 1.18 cm in middle marsh (MM), and 5.60 ± 0.89 ind/m^2^ and 7.67 ± 0.85 cm in high marsh (HM), respectively (Figures 1E–G). Compared with control soils, significant differences were observed in the soil physicochemical properties of crab-bioturbated soils (burrows and mounds) across the three salt marshes (Table 1). In HM, the pH, EC, SMC, TOC, and TN values were significantly elevated in both burrow and mound soils (all *p* < 0.05). Additionally, EC, SMC, and TOC were also higher in burrow and mound soils in MM (all *p* < 0.05). However, EC and TOC values were reduced in burrow and mound soils in LM (all *p* < 0.05). These results indicated that crab bioturbation exerted a considerable influence on soil physicochemical properties in various salt marshes.

**Table 1:** The soil properties after crab bioturbation in different salt marshes.

Soil	pH	EC (ms/cm)	SMC (%)	TOC (g/kg)	TN (mg/kg)	TP (mg/kg)
**Low marsh**	*p* = 0.174	*p* = 0.026	*p* = 0.222	*p* < 0.01	*p* = 0.292	*p* = 0.069
Control	8.55 ± 0.10a	5.98 ± 0.75a	32.75 ± 3.89a	11.79 ± 1.45a	554.64 ± 75.58a	541.52 ± 23.09a
Burrow	8.64 ± 0.06a	4.70 ± 0.57b	35.43 ± 0.85a	10.55 ± 1.13a	493.12 ± 65.55a	535.74 ± 7.68ab
Mound	8.65 ± 0.09a	4.99 ± 0.67b	33.70 ± 0.51a	7.63 ± 0.58b	486.18 ± 74.64a	515.06 ± 16.47b
**Middle marsh**	*p* = 0.924	*p* = 0.003	*p* < 0.01	*p* = 0.032	*p* = 0.476	*p* = 0.493
Control	8.71 ± 0.07a	4.05 ± 0.25a	22.38 ± 2.60a	3.69 ± 0.50ab	344.01 ± 67.93a	542.94 ± 20.07a
Burrow	8.70 ± 0.09a	4.99 ± 0.25b	28.50 ± 1.97b	4.56 ± 0.92a	370.92 ± 84.00a	560.29 ± 24.93a
Mound	8.68 ± 0.17a	5.26 ± 0.70b	29.38 ± 1.41b	3.25 ± 0.59b	401.44 ± 63.07a	544.80 ± 28.11a
**High marsh**	*p* = 0.005	*p* < 0.01	*p* = 0.019	*p* < 0.01	*p* = 0.01	*p* = 0.169
Control	8.29 ± 0.11a	4.62 ± 0.56a	19.36 ± 2.24a	4.83 ± 0.51a	386.35 ± 54.68a	563.08 ± 49.83a
Burrow	8.29 ± 0.17a	7.84 ± 0.59b	23.16 ± 1.23b	5.38 ± 0.36a	524.99 ± 51.80b	533.92 ± 23.73a
Mound	8.61 ± 0.13b	11.61 ± 1.64c	20.97 ± 1.78ab	6.52 ± 0.43b	470.07 ± 69.59b	510.24 ± 45.01a

### 3.2 Differences in abundant and rare subcommunity structures

**Figure 2:**
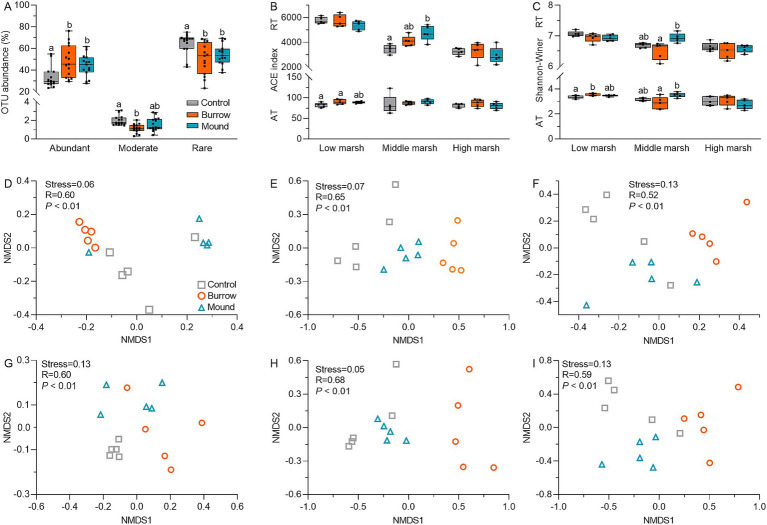
**(A)** The OTU abundances of abundant taxa (AT), moderate taxa (MT), and rare taxa (RT) in different salt marshes. The ACE **(B)** and Shannon-winner indices **(C)** of AT and RT subcommunities in different salt marshes. The NMDS analysis of AT **(D–F)** and RT **(G–I)** subcommunities in different salt marshes.

We obtained 2,698,112 high-quality reads (376 bp per read), with a median read count of 59,958 per sample (range: 39,007–73,900) in 45 samples. After subsampling each sample to minimum sequencing depth, the reads were clustered into 12,052 OTUs. The rarefaction curves of Sobs and Shannon–Wiener index plateaued with increasing OTUs, and all Good’s coverages exceeded 90%, suggesting the observed OTUs were sufficient for further analysis (Supplementary Figure S1). Based on the cutoffs of OTU relative abundance (1 and 0.01%), all OTUs were categorized into abundant taxa (102 OTUs, 41.87%) moderate taxa (7 OTUs, 1.56%), and rare taxa (11,943 OTUs, 56.67%) (Figure 2A), indicating the bacterial communities were mainly composed of abundant taxa (AT) and rare taxa (RT). Compared with controls, both AT and RT subcommunity α-diversities, as indicated by ACE and Shannon–Wiener indices, differed in crab-bioturbated soils (mounds and burrows), particularly in MM (all *p* < 0.05) (Figures 2B,C). Moreover, NMDS analysis showed significant differences in β diversity between crab-bioturbated and control soils for AT and RT subcommunities in the salt marshes (all stress <0.15 and *p* < 0.01) (Figures 2D–I). These results suggested that crab bioturbation altered both α and β diversity of AT and RT subcommunities.

**Figure 3:**
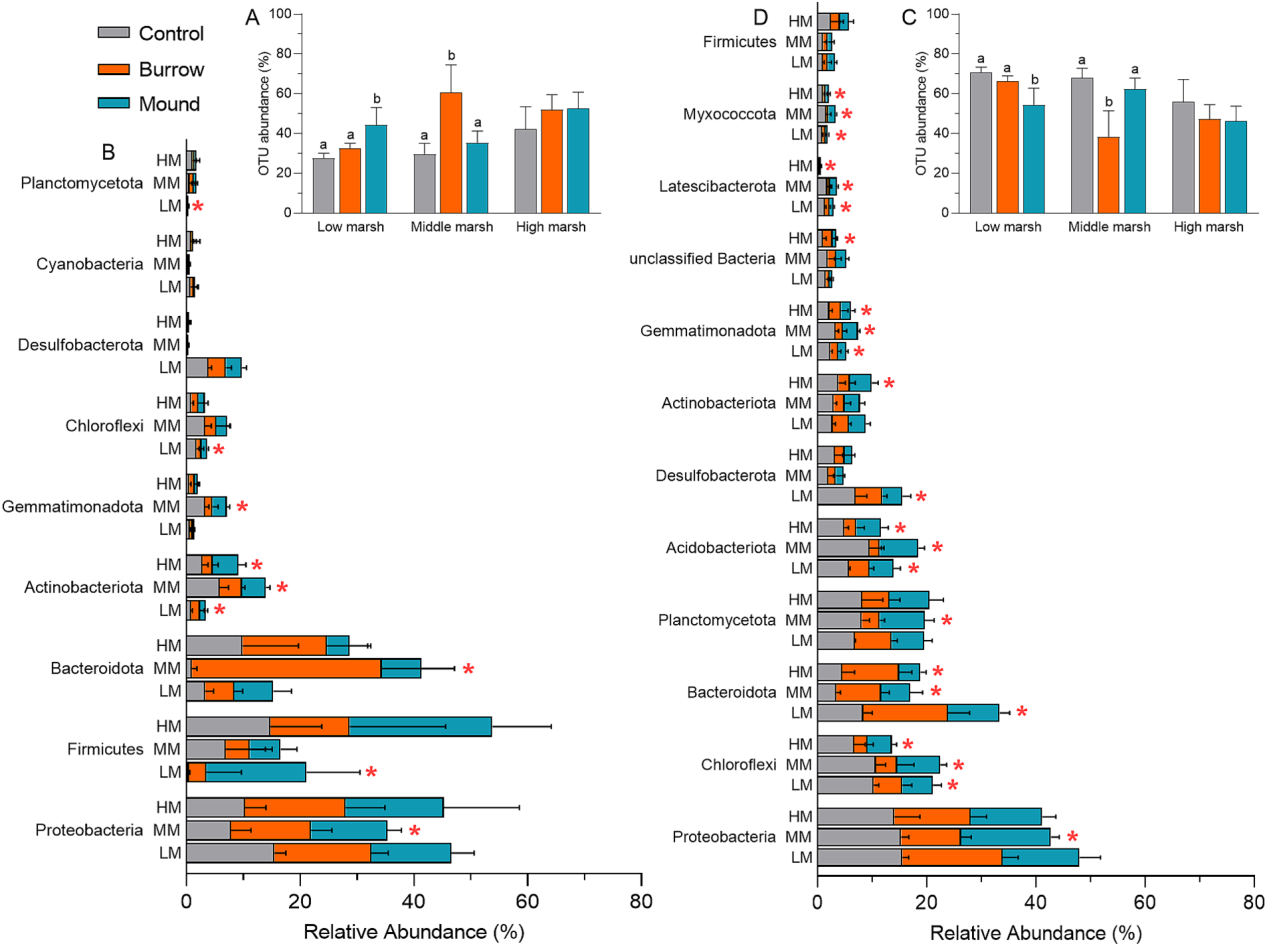
**(A,C)** The OTU abundances of AT and RT subcommunities in different salt marshes. **(B,D)** Dominant bacteria of AT and RT subcommunities in different salt marshes at phylum level. The red asterisks (*) indicate the significantly different bacteria. The same is shown below.

Compared with controls of AT subcommunities, OTU abundances were notably higher in crab-bioturbated soils in LM and MM (Figure 3A). Generally, AT-OTUs belonged to Proteobacteria (14.14%), Firmicutes (10.16%), Bacteroidota (9.47%), Actinobacteriota (2.95%), Gemmatimonadota (1.16%), Chloroflexi (1.57%), Desulfobacterota (1.17%), Planctomycetota (0.43%), and Cyanobacteria (0.38%) with a total of 41.02%. There were several phyla with significantly different abundances in between crab-bioturbated soils (burrows and mounds) and controls (Figure 3B). For RT subcommunities, OTU abundances in crab-bioturbated soils were lower than them in controls (Figure 3C). The RT subcommunities mainly comprised 12 phyla: Proteobacteria (14.65%), Firmicutes (1.29%), Bacteroidota (7.68%), Actinobacteriota (2.95%), Gemmatimonadota (2.08%), Chloroflexi (6.36%), Desulfobacterota (2.95%), Planctomycetota (6.62%), Acidobacteriota (4.89%), unclassified Bacteria (1.27%), Latescibacterota (0.80%), and Myxococcota (0.81%) with a total of 52.35%. It was observed that many phyla were markedly reduced in crab-bioturbated soils compared with controls (Figure 3D). Specifically, there were 6, 7, and 5 phyla with decreasing abundances in crab-bioturbated soils of LM, MM and HM, respectively.

**Figure 4:**
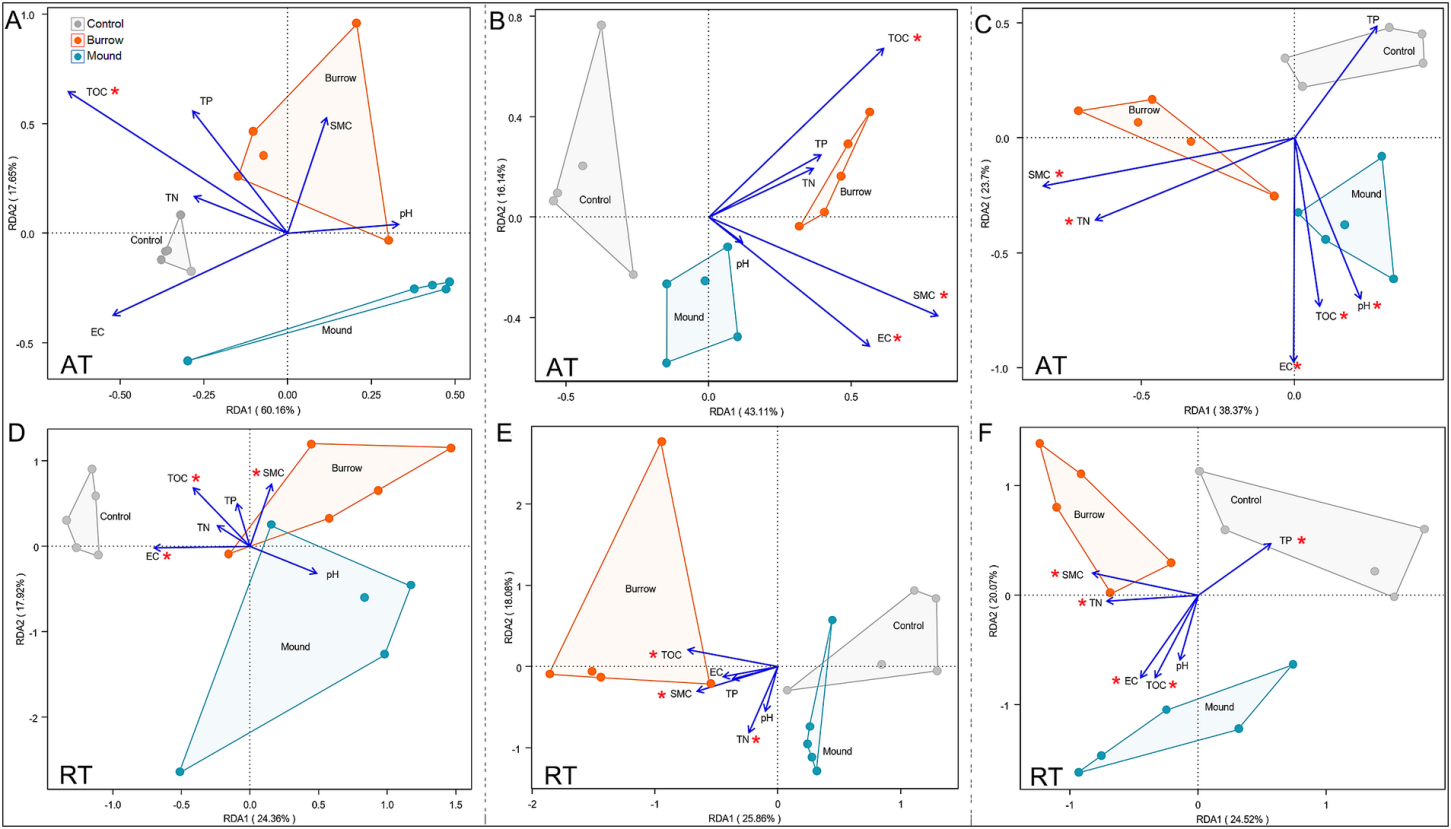
Redundancy analysis (RDA) between soil properties and AT **(A–C)** and RT **(D–F)** subcommunities at phylum level in different salt marshes.

Furthermore, we performed RDA to determine the main explanatory variables influencing the differences in AT and RT subcommunity structures. In LM, AT subcommunities were significantly associated with TOC (*R*^2^ = 0.684) (Figure 4A), while RT subcommunities were related to TOC (*R*^2^ = 0.645), EC (*R*^2^ = 0.478), and SMC (*R*^2^ = 0.557) (Figure 4D). In MM, bacterial subcommunities were significantly correlated with SMC (AT: *R*^2^ = 0.646, RT: *R*^2^ = 0.505), TOC (AT: *R*^2^ = 0.640, RT: *R*^2^ = 0.514), EC (AT: *R*^2^ = 0.502), and TN (RT: *R*^2^ = 0.701) (Figures 4B,E). Moreover, almost all soil factors were significantly associated with AT and RT subcommunities in HM (all *p* < 0.05), with the correlation coefficients ranging from 0.492 to 0.873 (Figures 4C,F). Among these, the results showed EC was the most important factor for AT (*R*^2^ = 0.873) and RT (*R*^2^ = 0.747) subcommunities.

### 3.3 Responses of bacterial co-occurrence networks to crab bioturbation

**Figure 5:**
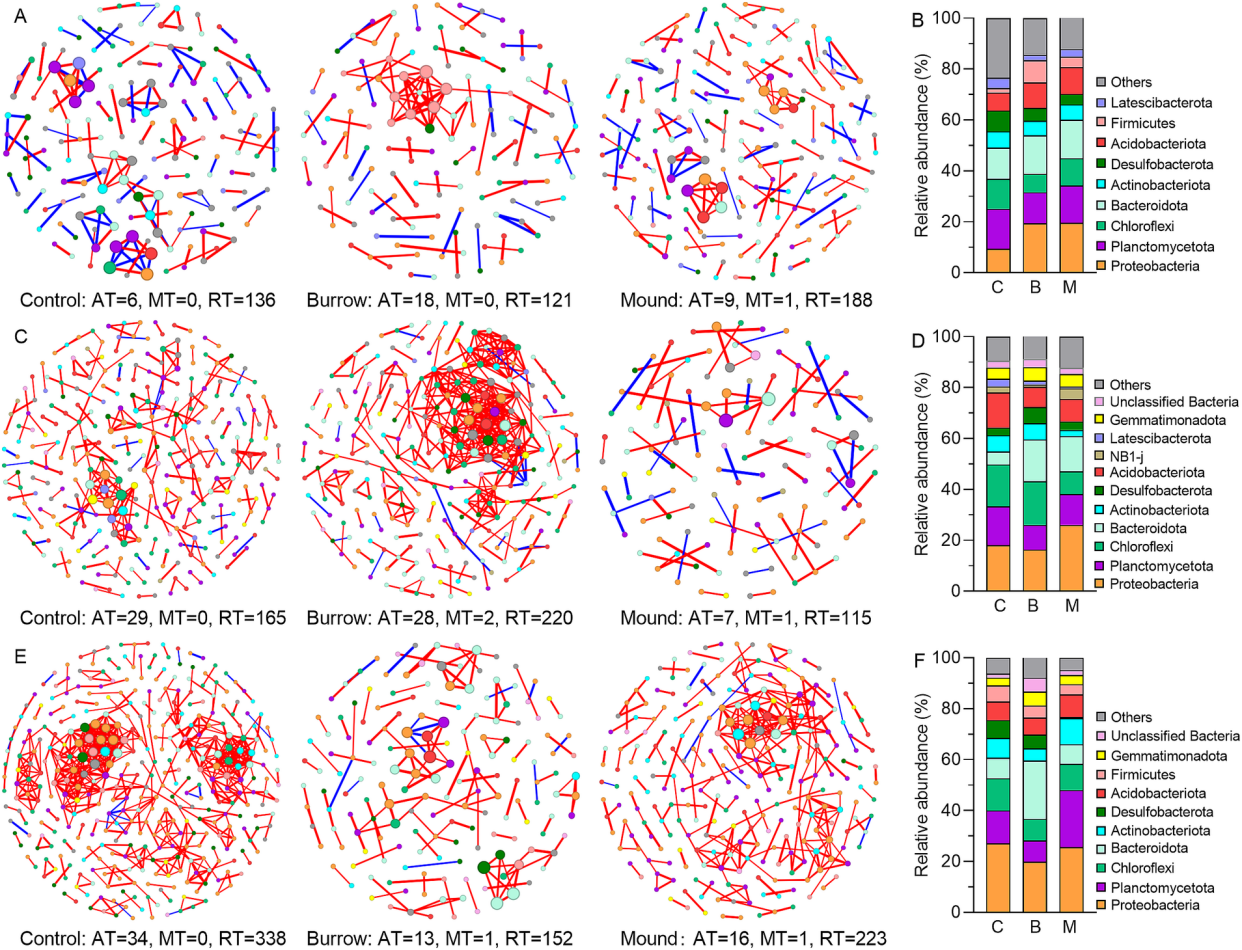
Bacterial co-occurrence networks and dominant bacterial phylum of different soils in low marsh **(A,B)**, middle marsh **(C,D)**, and high marsh **(E,F)**. The red lines indicate positive relationships between OTUs, and the blue lines indicate negative relationships between OTUs.

Our study constructed nine co-occurrence networks to evaluate the effect of crab bioturbation on bacterial interaction relationships. Each co-occurrence network degree followed a power-law distribution (all *R*^2^ > 0.98), suggesting all community networks were scale-free and non-random. As showed in Table 2, the topological properties (ACC, APL, and MD) of empirical networks were higher than those of Erdös–Rényi random networks, indicating nine empirical networks exhibited clear small-world properties. Compared with control networks, the small-world coefficients have changed with varying degrees in burrows and mounds in the three salt marshes. Moreover, the OTU relationships were also different in crab bioturbated networks in each salt marsh, particularly in LM. Notably, the comparisons of control bacterial networks among LM, MM and HM showed certain differences in topological properties and dominant bacteria (Table 2; Figure 5). These differences might be influenced by various abiotic and biotic factors, such as soil physicochemical properties, plant selections and bacterial relationships.

**Table 2:** The empirical and random bacterial co-occurrence networks in different soils of three salt marshes.

Samples	Node	Empirical networks							Random networks				Small-world coefficient
		Edge	ACC	APL	MD	PL	NL	Power (*R*^2^)	ACC	SD	APL	MD	
Low marsh													
Control	171	136	0.849	1.588	0.956	57.35%	42.65%	0.985	0.009	0.029	7.805	0.768	463.65
Burrow	139	102	0.683	1.585	0.885	80.39%	19.61%	0.999	0.012	0.026	7.409	0.798	266.05
Mound	198	130	0.804	1.135	0.975	70.77%	29.23%	0.998	0.008	0.027	8.132	0.848	720.06
Middle marsh													
Control	297	279	0.563	2.934	0.929	92.47%	7.53%	0.998	0.007	0.019	7.641	0.774	209.46
Burrow	250	464	0.591	5.185	0.707	95.69%	4.31%	0.994	0.015	0.019	4.282	0.53	38.33
Mound	123	69	0.667	1.093	0.976	69.57%	30.43%	0.999	0.009	0.028	4.842	0.891	328.31
High marsh													
Control	372	680	0.629	3.88	0.828	97.65%	2.35%	0.991	0.01	0.02	4.633	0.534	75.11
Burrow	166	129	0.702	1.3	0.966	89.92%	10.08%	0.996	0.01	0.025	7.837	0.789	423.20
Mound	250	295	0.557	3.752	0.864	96.95%	3.05%	0.995	0.01	0.018	5.995	0.676	89.00

Each and every bacterial co-occurrence network was made up of a large number of RT-OTUs, ranging from 85.05 to 95.77% (Figure 5). At the phylum level, several bacterial abundances exhibited significant variations in crab-bioturbated networks when compared with those of the controls. For instance, in the LM, Proteobacteria and Firmicutes showed such changes; in the MM, it was Actinobacteriota; and in the HM, Planctomycetota and Bacteroidota exhibited significant alterations. Moreover, the topological properties (module, betweenness centrality, degree centrality, etc.) were also modified by crab bioturbation in each marsh (Supplementary Tables S1–S9). In particular, the core modules (with OTU counts greater than 4) and key OTUs (having high betweenness centrality of no less than 10) displayed significant differences in crab-bioturbated networks as opposed to the controls’ (Supplementary Figure S2). Specifically, in MM (Supplementary Tables S4–S6), there were eight core modules (comprising 74 key OTUs) in the burrow network and only one core module (with no key OTUs) in the mound’s network, in contrast to 11 core modules (containing 31 key OTUs) in the control’s network. Similarly, in HM (Supplementary Tables S7–S9), there were five core modules (with no key OTUs) in the burrow’s network and nine core modules (comprising 54 key OTUs) in the mound’s network, compared to 12 core modules (containing 102 key OTUs) in the control’s network.

### 3.4 Responses of AT and RT subcommunity assembly processes to crab bioturbation

**Figure 6:**
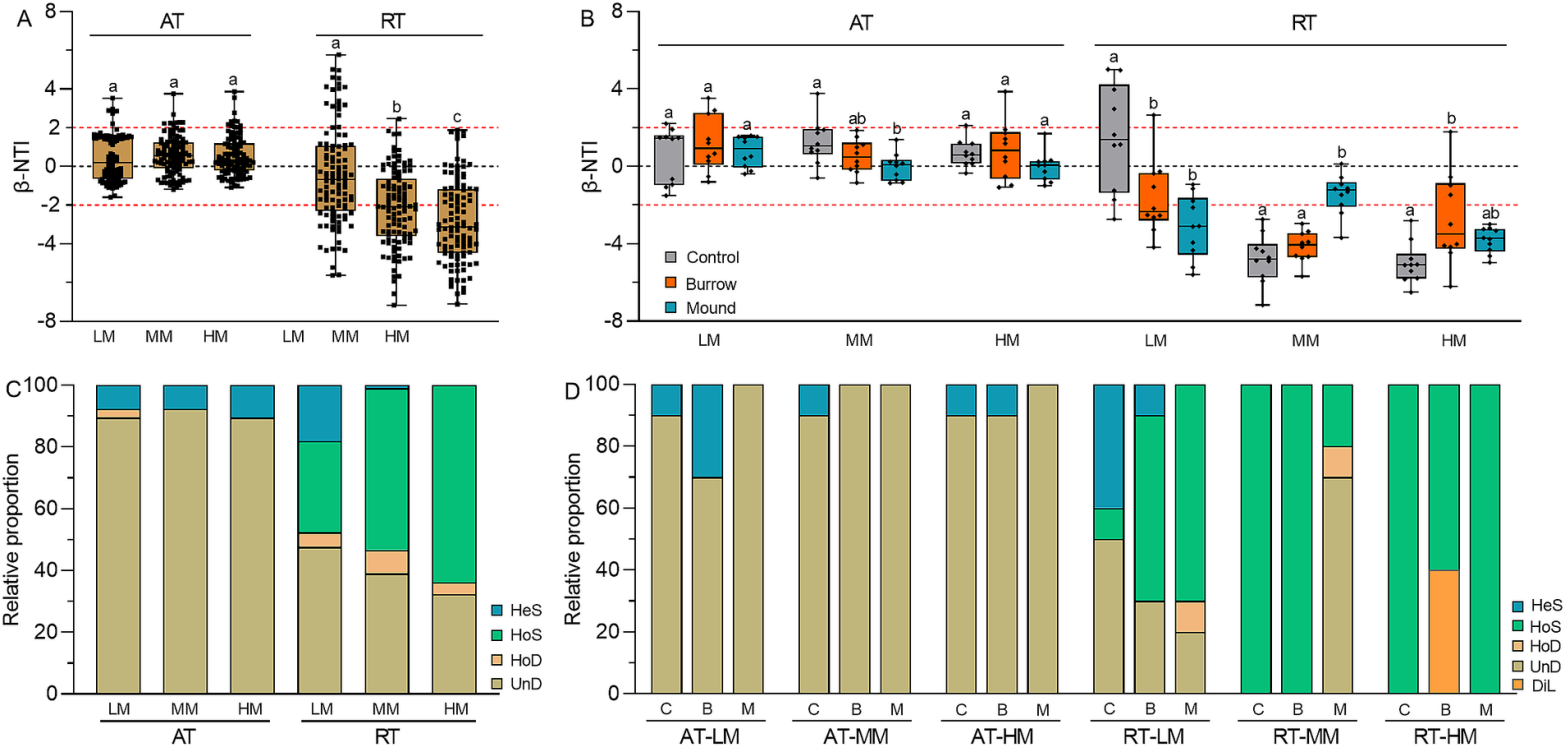
**(A)** β-NTI values of AT and RT subcommunities in different salt marshes. **(B)** β-NTI values of AT and RT subcommunities in control, burrow, and mound soils. **(C)** Relative proportions of assembly processes in AT and RT subcommunities in different salt marshes. **(D)** Relative proportions of assembly processes in AT and RT subcommunities in control, burrow, and mound soils. HeS represents heterogeneous selection, HoS represents homogeneous selection, HoD represents homogenizing dispersal, DiL represents dispersal limitation, and UnD represents undominated process, respectively.

We used β-NTI to quantify the bacterial community assembly of stochastic and deterministic processes. The results showed the β-NTIs of AT subcommunities greatly fell within the range of −2 to 2, suggesting the AT subcommunity assemblies were mainly dominated by stochastic processes with the proportion of 92.38% in LM, 92.38% in MM and 89.52% HM (Figure 6A). Meanwhile, the β-NTIs of RT subcommunities exhibited broad distributions ranging from −7.17 to 5.76 (*p* < 0.05). This result indicated the RT subcommunity assemblies were shaped by both stochastic and deterministic processes, accounting for 52.38 and 47.62% in LM, 46.67 and 53.33% in MM, and 37.14 and 62.86% in HM, respectively. Moreover, crab bioturbation had different influence on the assemblies of AT and RT subcommunities. Compared with controls, the β-NTI changes of burrows and mounds were minor in AT subcommunities but pronounced in RT subcommunities, suggesting crab bioturbation greatly influenced RT subcommunity assemblies in each salt marsh (Figure 6B). Additionally, RC_Bray_ analysis showed the assembly process of AT subcommunities was mainly governed by UnD (LM: 89.52%, MM: 92.38%, HM: 89.52%), while the assembly processes of RT subcommunities were varying extend influenced by UnD, HoD, HoS and HeS (Figure 6C). In different salt marshes, compared with controls, there was no obvious change of UnD in AT subcommunity assembly of crab bioturbated soils, but more variable in RT subcommunity assembly with greater proportions of HoS in LM, UnD in MM, DiL in HM, respectively (Figure 6D). All the results obtained indicated that crab bioturbation significantly altered the assemblies of RT subcommunities but had a slight influence on the AT subcommunity assemblies.

## 4 Discussion

### 4.1 Effects of crab bioturbation on soil physicochemical properties

Crab burrows act as dynamic conduits for the exchange of matter and energy within intertidal marshes and have garnered increasing scientific interest (Thomas and Blum, 2010; Li et al., 2021; Sudharaka et al., 2023; Xiao et al., 2024). Our field investigation found the intensity of crab bioturbation, correlated with diverse diameters and densities of crab burrows across the entire intertidal marsh of the Yellow River estuary (Figure 1). Previous studies have demonstrated that the soil water content and salinity are key environmental factors influencing crab species distribution and habitat selection (Li et al., 2017; Chen X. et al., 2022). Our study observed that *M. japonicus* was more prevalent in low and middle marshes, whereas *H. tientsinensis* was dominant in middle and high marshes, characterized by higher densities and wider diameters of crab burrows. It has been reported that crab species exhibit a positive linear relationship between carapace size and width, and the direct parameters of their burrows (El-Hacen et al., 2019; Xie et al., 2020).

Our study confirmed that crab bioturbation redistributes soil nutrients and water-salt transportation (Table 1), in line with previous findings (Wang et al., 2010; Qiu et al., 2019; Forgeron et al., 2021). Notably, soil organic matter, water content, and salinity differed significantly between crab-bioturbated and control soils in the three salt marshes. More frequent tidal flushing carries away substantial amounts of soil nutrients and salinity from low marshes (Xiao et al., 2020; Chen X.G. et al., 2022), potentially leading to reduced soil organic matter, total phosphorus, and soil salinity in crab-bioturbated soils. In contrast, when tidal flushing is less frequent, crab burrows can retain plant residues and accumulate soil salinity through water evaporation (Thomas and Blum, 2010; Sudharaka et al., 2023). Consistent with our findings, the organic matter and salinity contents of crab-bioturbated soils were higher than those of controls in middle and high marshes.

### 4.2 Effects of crab bioturbation on the structure and co-occurrence network of abundant and rare subcommunities

The rare subcommunity constitutes an important pool of diverse functions, capable of generating specific soil functions and mitigating environment disturbances through functional redundancy (Li Y.B. et al., 2023; Li J. et al., 2023; Yao et al., 2019; Yang et al., 2022). Our study focused on the changes in abundant and rare bacterial subcommunities within crab-bioturbated soils. A significant portion of OTUs (over 99%) clustered into rare subcommunity, accounting for approximately 50% of the total OUT abundance. We found that crab bioturbation significantly altered the diversity and composition of the rare subcommunity (Figures 2, 3). Particularly in low and middle marshes, overall bacterial community diversity decreased alongside the decline in rare subcommunity diversity in crab-bioturbated soils. This supports the notion that rare bacteria are crucial for maintaining the entire bacterial community diversity (Lynch and Neufeld, 2015; Jia et al., 2018; Wang et al., 2023). Moreover, both abundant and rare subcommunities exhibited clear changes in crab-bioturbated soils compared with control soils (Figure 3). Specifically, four abundant bacteria (Proteobacteria, Firmicutes, Bacteroidota, and Actinobacteriota) increased in the relative abundance, whereas seven rare bacteria (Chloroflexi, Bacteroidota, Acidobacteriota, Gemmatimonadota, Latescibacterota, Myxococcota, and Desulfobacterota) decreased in crab-bioturbated soils.

Consistent with a previous study (Wu et al., 2020), our finding confirmed that crab bioturbation substantially modified bacterial co-occurrence networks in salt marshes. The topological properties of small-world and modularity were significantly weakened in crab-bioturbated bacterial co-occurrence networks compared with controls’ (Table 2; Figure 5). These changes may reduce network complexity and connectivity, thereby impairing bacterial community stability and functional traits (Butler and O’Dwyer, 2018; Gao et al., 2022). Additionally, we found that crab bioturbation enhanced positive correlations among bacterial relationships, which may increase the vulnerability of bacterial community, as the extinction of some bacteria could lead to the reduction or extinction of positively correlated bacteria through feedback loops (Faust, 2021; Li Y.B. et al., 2023; Li J. et al., 2023). Notably, it was found that rare bacteria play a significant role in the internal relationships of co-occurrence networks (Figure 5; Supplementary Tables S1–S9). For example, as much as 29.78, 54.03, 47.00 and 43.24% of RT-OTUs had high betweenness centrality (having high betweenness centrality of no less than 10) in the core modules of MM-control’s, MM-burrow’s, HM-control’s and HM-mound’s respectively, which suggests that rare bacteria are crucial nodes for information connectivity within co-occurrence networks. In particular, the betweenness centrality of five RT-OTUs surpassed 1,000 in MM-burrow’s.

### 4.3 Effects of crab bioturbation on assembly processes of abundant and rare subcommunities

Elucidating the mechanisms underlying community assembly is pivotal for comprehending soil microbial diversity and functionality within the realm of microbial ecology (Byrnes et al., 2014; Escalas et al., 2019; Yang, 2021). In the intertidal wetland, the assembly of the soil microbial community was subject to a wide variety of abiotic and biotic factors, among which were soil physicochemical properties, tide movements, bacterial traits, and plant species, among others. Our study has uncovered significant alterations in the assembly processes of both abundant and rare subcommunities after crab bioturbation (Figures 6A,B). Typically, the assemblies of abundant subcommunities were primarily driven by stochastic processes, while the rare subcommunity assemblies were largely regulated by both deterministic and stochastic processes. This finding aligns with several previous reports concerning bacterial community assembly in coastal wetlands (Liu et al., 2015; Gao et al., 2020; Yang et al., 2022; Mohapatra et al., 2023). Specifically, our results demonstrated that the assemblies of abundant subcommunities were predominantly affected by the undominant process, irrespective of crab bioturbation. However, crab bioturbation modified the contributions of undominated processes and homogeneous selection in the assembly of rare subcommunities. On the one hand, crab burrows serve as active conduits for both seawater and microorganisms (Wu et al., 2020; Xie et al., 2020; Tongununui et al., 2021). This might facilitate stochastic exchanges of abundant bacteria through pore water flux, particularly in low and middle salt marshes, with the aid of frequent tidal movements. On the other hand, certain abundant bacteria (such as Acidobacteriota, Proteobacteria, and Chloroflexi) possess high migration rates, assisted by species traits like flagella, cell size, and resistance, which strengthen undominant diffusion (Luan et al., 2020). Conversely, rare bacteria are more susceptible to environmental pressures, and maladaptations can result in the extinction of larger rare species, leaving fewer surviving bacteria (Dini-Andreote et al., 2015; Xun et al., 2019). For instance, soil salinity and nutrients act as powerful environmental filters for soil bacterial communities in coastal wetlands (An et al., 2019; Zhang et al., 2020; Li Y.B. et al., 2023; Li J. et al., 2023), presenting significant challenges to the assembly and diversity of rare subcommunities. Moreover, it is notable that when comparing the three salt marshes, plant selection has an impact on the assembly of RT subcommunities rather than that of AT subcommunities. This might be closely associated with vegetation litters that possess different organic matter compositions in terms of cellulose, hemicellulose, and lignin (Nemergut et al., 2013; Ren et al., 2018). Specifically, *Spartina alterniflora*, which was dominant in LM, contained higher contents of cellulose, while *Tamarix chinensis*, dominant in HM, contained more lignin. Some researches have shown that rare bacteria contribute to a diverse pool of functions, which results in their different abilities to utilize these carbon sources (Jiang et al., 2020; Wang et al., 2023).

## 5 Conclusion

In this study, we examined the structures and assembly processes of abundant and rare bacterial subcommunities in intertidal marsh soils in response to crab bioturbation across different salt marshes. Our findings provide compelling evidence that crab bioturbation significantly altered the structures of rare subcommunities, whereas the structures of abundant subcommunities remained relatively unaffected. At phylum level, the relative abundances of many rare bacteria were notably reduced in crab-bioturbated soils compared with controls, while the relative abundances of abundant bacteria exhibited no discernible changes. Notably, rare bacteria played an important role in bacterial co-occurrence networks and were also influenced by crab bioturbation. Furthermore, the responses of abundant and rare subcommunity assemblies to crab-bioturbation differed in the three salt marshes. Particularly for rare subcommunities, crab bioturbation markedly increased the significance of the homogeneous selection process in low marsh, the undominated process in middle marsh, and dispersal limitation in high marsh. In summary, we propose that crab bioturbation substantially modified the soil bacterial community structure and co-occurrence network in the intertidal marsh, as the assembly processes of abundant and rare subcommunities exhibited distinct responses to crab bioturbation across different salt marshes.

## Data Availability

The datasets presented in this study can be found in online repositories. The names of the repository/repositories and accession number(s) can be found at: https://www.ncbi.nlm.nih.gov/, PRJNA1114880.

## Footnotes

^1^ https://www.arb-silva.de/

^2^ https://sourceforge.net/projects/rdp-classifier/

## Supplementary material

SUPPLEMENTARY FIGURE S1 Rarefaction curves of Sobs **(A)** and Shannon-Wiener **(B)** at OTU level in different soil bacterial communities. **(C)** Good’s coverages of bacterial communities in different salt marshes.

SUPPLEMENTARY FIGURE S2 The distributions of core modules (OTU counts > 4) **(A–C)** and key OTUs (high betweenness centrality ≥ 10) **(D–F)** in different bacterial co-occurrence networks of low, middle, and high marshes.
