# Supplementary figures and images for: Crab bioturbation alters the community assemblies of abundant and rare bacteria on an intertidal wetland in the Yellow River estuary
Source: Front Microbiol. 2025 Jan 31;16:1521363. doi: 10.3389/fmicb.2025.1521363 (PMC11826314; doi:10.3389/fmicb.2025.1521363)

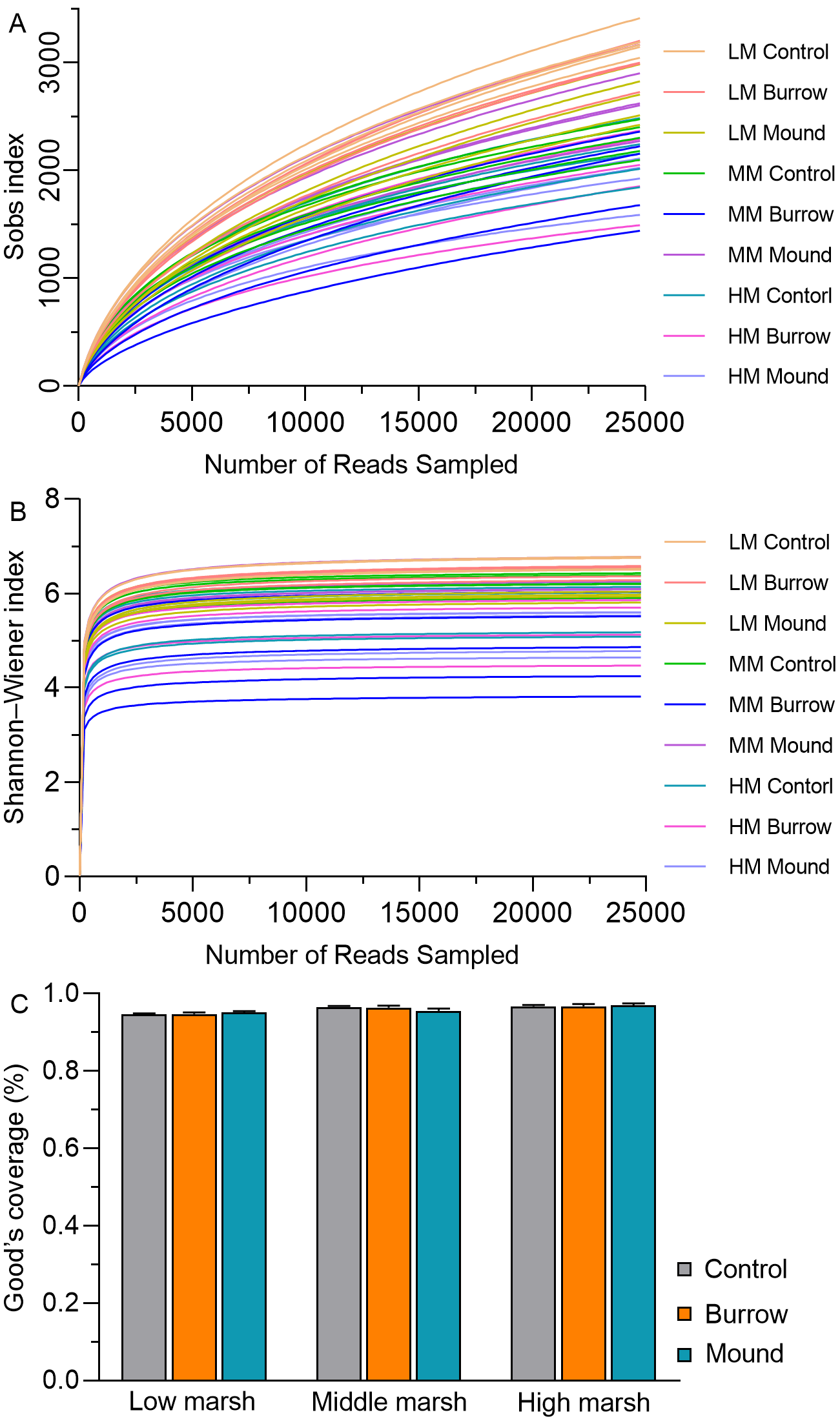

Supplement: SUPPLEMENTARY FIGURE S1 — Rarefaction curves of Sobs (A) and Shannon-Wiener (B) at OTU level in different soil bacterial communities. (C) Good’s coverages of bacterial communities in different salt marshes. [file Image_1.tif]

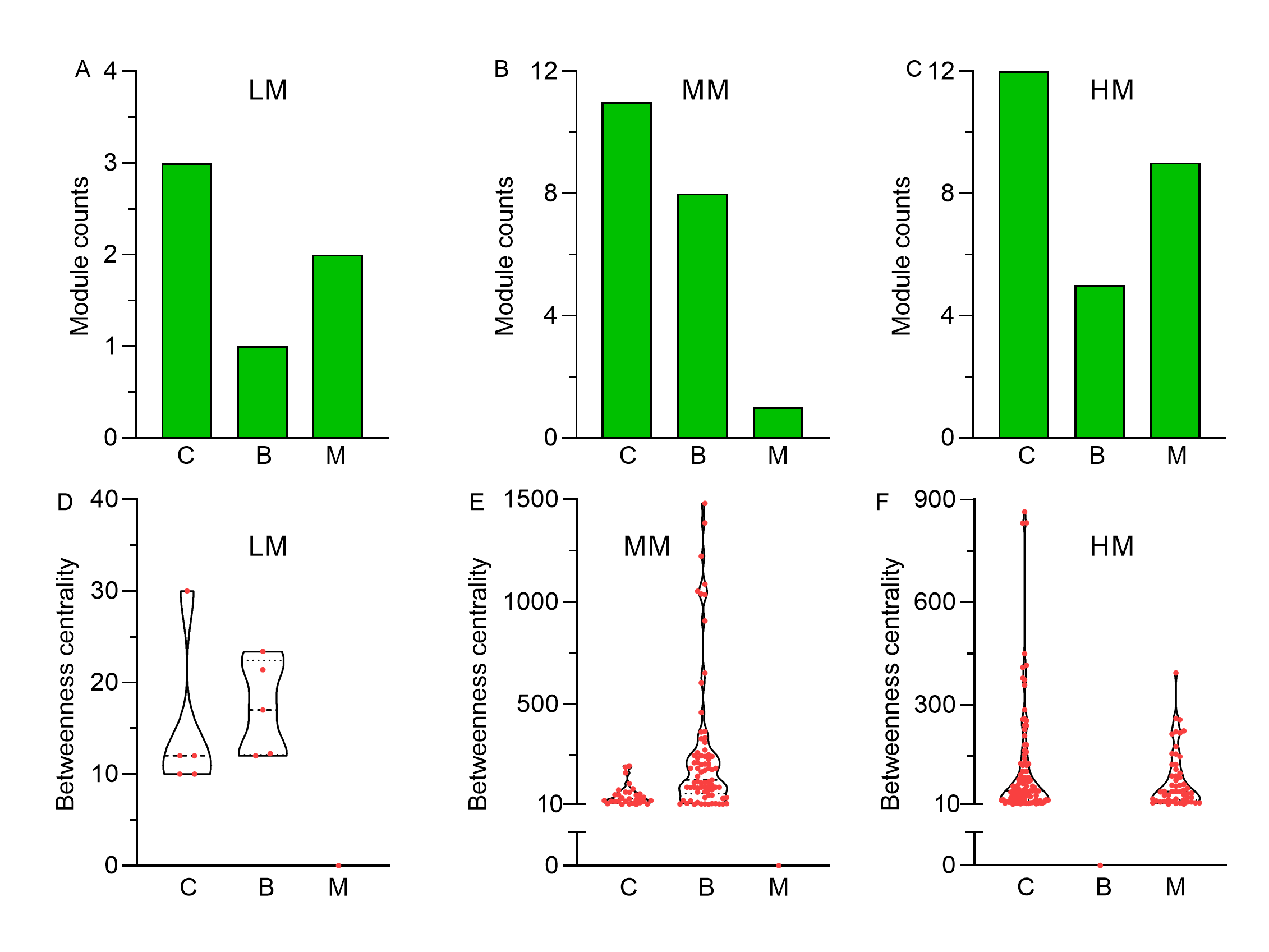

Supplement: SUPPLEMENTARY FIGURE S2 — The distributions of core modules (OTU counts > 4) (A–C) and key OTUs (high betweenness centrality ≥ 10) (D–F) in different bacterial co-occurrence networks of low, middle, and high marshes. [file Image_2.tif]
